# Out of Thin Air: Microbial Utilization of Atmospheric Gaseous Organics in the Surface Ocean

**DOI:** 10.3389/fmicb.2015.01566

**Published:** 2016-01-20

**Authors:** Jesús M. Arrieta, Carlos M. Duarte, M. Montserrat Sala, Jordi Dachs

**Affiliations:** ^1^Division of Biological and Environmental Science and Engineering, Red Sea Research Center, King Abdullah University of Science and TechnologyThuwal, Saudi Arabia; ^2^Department of Global Change Research, Institut Mediterrani d'Estudis Avançats, Consejo Superior de Investigaciones Científicas/Universitat de les Illes BalearsEsporles, Spain; ^3^Departament de Biologia Marina i Oceanografia, Institut de Ciències del Mar, Consejo Superior de Investigaciones CientíficasBarcelona, Spain; ^4^Department of Environmental Chemistry, Institute for Environmental Assessment and Water Research, Consejo Superior de Investigaciones CientíficasBarcelona, Spain

**Keywords:** air–sea exchange, gas-phase organics, GOC, microbial carbon demand, ocean

## Abstract

Volatile and semi-volatile gas-phase organic carbon (GOC) is a largely neglected component of the global carbon cycle, with poorly resolved pools and fluxes of natural and anthropogenic GOC in the biosphere. Substantial amounts of atmospheric GOC are exchanged with the surface ocean, and subsequent utilization of specific GOC compounds by surface ocean microbial communities has been demonstrated. Yet, the final fate of the bulk of the atmospheric GOC entering the surface ocean is unknown. Our data show experimental evidence of efficient use of atmospheric GOC by marine prokaryotes at different locations in the NE Subtropical Atlantic, the Arctic Ocean and the Mediterranean Sea. We estimate that between 2 and 27% of the prokaryotic carbon demand was supported by GOC with a major fraction of GOC inputs being consumed within the mixed layer. The role of the atmosphere as a key vector of organic carbon subsidizing marine microbial metabolism is a novel link yet to be incorporated into the microbial ecology of the surface ocean as well as into the global carbon budget.

## Introduction

**Figure 1:**
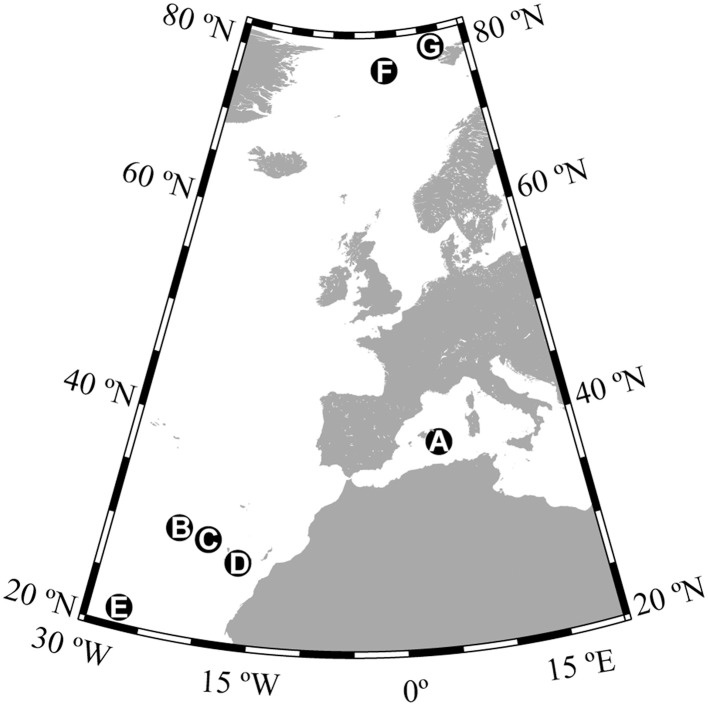
**Locations where sampling and experiments were conducted**. The letters A–G correspond to the station names used throughout the manuscript.

Global sources of gaseous organic compounds include natural emission form forests and oceans, natural and anthropogenic biomass burning, the production, and use of fossil fuels and other industrial activities (Hewitt, 1999). Gas-phase organic carbon (GOC) compounds are removed from the atmosphere mainly by photochemical degradation (Atkinson and Arey, 2003) and deposition into terrestrial (Park et al., 2013) and oceanic ecosystems (Dachs et al., 2005). In fact, the global ocean is a major sink for atmospheric carbon, including CO_2_ (Takahashi et al., 2002; Sabine et al., 2004), black carbon, aerosol-bound organic carbon, and organic carbon delivered with wet deposition (Jurado et al., 2008). The oceans are also a likely sink for gas-phase anthropogenic and biogenic organic compounds (Jurado et al., 2005), as supported by reports of large diffusive inputs of GOC in surface waters of the subtropical Atlantic, exceeding atmosphere-ocean carbon exchanges of dry aerosol deposition and net CO_2_ exchange (Dachs et al., 2005). The deposition of atmospheric organic compounds has been suggested as a potential carbon source for microbial metabolism in oligotrophic and unproductive ocean ecosystems (del Giorgio and Cole, 1998). Some GOC compounds like methanol, acetone, or acetaldehyde have been shown to be consumed rapidly by marine microbes in the surface ocean (Dixon and Nightingale, 2012; Dixon et al., 2013), supporting a significant fraction of the bacterial carbon demand in surface waters at some locations (Dixon et al., 2011). However, atmospheric GOC is a complex mixture containing many other compounds that have been rarely, if ever, directly measured (Goldstein and Galbally, 2007) many of which can be exchanged with the ocean (Hauser et al., 2013), which suggests that air–sea fluxes of organic molecules must be much larger than calculated from surveys of the a few abundant components of the atmosphere. In fact, the fluxes of the most frequently characterized GOC compounds like methanol, acetone, and acetaldehyde estimated for the central Atlantic (Yang et al., 2014) are orders of magnitude lower than the bulk estimates of net air–sea exchange of total organic gas-phase compounds (Dachs et al., 2005). These large differences suggest that a major fraction of the organic molecules exchanged between the ocean and the atmosphere may be missed by current compound-specific surveys (Yang et al., 2014). We hypothesized that many of these non-characterized components of GOC entering the ocean may also be available to microbial consumption and thus, that atmospheric inputs of GOC may sustain a much larger fraction of the prokaryotic carbon demand in surface waters than hitherto expected. In this paper, we present the first estimates of prokaryotic utilization of ambient levels of atmospheric gas-phase organic materials equilibrated with surface seawater. Seven experiments were carried out at different locations (Figure 1), two of them in the Arctic Ocean, one in the Mediterranean Sea, and four in the NE Subtropical Atlantic, where large inputs of atmospheric GOC to the ocean have been reported (Dachs et al., 2005).

## Materials and methods

### Sampling locations and GOC flux estimates

GOC and microbial communities were sampled at seven different locations in the Mediterranean Sea, the Subtropical Atlantic Ocean and the Arctic Ocean (Figure 1). GOC concentrations in seawater were estimated, as described elsewhere (Dachs et al., 2005) indirectly by equilibrating air and water for 30 min by bubbling pre-filtered air (QMA filter, Whatman) through 50 mL of acidified (H_3_PO_4_, pH 1–2) water (HPLC grade water, Merck) upwind of the research vessel. The resulting levels of GOC dissolved in seawater are given by GOC/*H*′, where GOC is the concentration of volatile and semi-volatile organics in the atmosphere and *H*′ is the dimensionless Henry's Law constant defined as the ratio between vapor pressure over aqueous solubility (Lakaschus et al., 2002). Only a fraction of GOC comprising mainly semi-volatile compounds with low *H*′-values is effectively retained in seawater, while the more volatile compounds have the tendency to partition to the atmosphere. Thus, the relative composition of the atmospheric GOC and that of the GOC entering the ocean are different but we will refer to both of them as GOC throughout the manuscript for ease of reading. After sampling, the water was immediately transferred into pre-combusted glass ampoules (450°C, 6 h) and sealed. The total organic carbon (TOC) concentration in the HPLC grade water representing GOC/*H*′ as the GOC equilibrated in water was determined in duplicate by high temperature catalytic oxidation on a Shimadzu TOC-5000A. Standards provided by Dennis A. Hansell and Wenhao Chen (University of Miami, USA) of 44–45 μmol dissolved organic carbon (DOC) and 2 μmol TOC were used to assess the accuracy of the estimates. Purging of samples prior to injection was not performed as inorganic carbon had been already evacuated during sampling.

Surface waters can be a source or a sink of atmospheric gaseous carbon depending on the relative concentrations of the molecules involved present in air and surface seawater. Thus, in order to calculate the net diffusive exchange of gaseous organic molecules, we needed an estimation of the fraction of exchangeable dissolved organic carbon (EDOC), defined as the amount of organic carbon in surface waters that can be exchanged with the atmosphere. EDOC was determined by purging 1 L of seawater collected at 5 m depth with pure N_2_ (grade 5.0) for 5–8 min and equilibrating the outgassing N_2_ products in 40 mL of pure acidified (H_3_PO_4_, pH 1–2) water (HPLC grade water, MERCK). Water was then transferred to pre-combusted ampoules and analyzed for organic carbon content as described above. The efficiency of EDOC extraction by this procedure is 53 ± 28 and 80 ± 26% as determined using acetone and toluene standards (Dachs et al., 2005). GOC and EDOC concentrations were corrected for field blanks consisting of acidified water subjected to the same manipulations except bubbling. Field blanks for GOC ranged from 15 to 35 μM, in good agreement with the theoretical amount of CO_2_ expected to dissolve in acidified seawater in contact with the atmosphere (12 μM at 298°K and 30 μM at 273°K).

Diffusive air–sea OC exchange was estimated using the wind speed dependence of the mass transfer velocity (*k*_600_) from instantaneous wind speeds (*U*_10_, m s^−1^) following the equation $k_{600}=0.24U_{10}^{2}+0.061U_{10}$ (Nightingale et al., 2000). Using other published parameterisations of the relationship between *k*_600_ and wind speed did not change the direction of the observed fluxes and resulted usually in minor changes in the magnitude of the net flux calculated (Supplementary Materials).

Net diffusive fluxes of organic carbon (*F*_*OC*_) were estimated as the sum of gross volatilization ($F_{OC,VOL}=k^{\prime }\times \text{EDOC}$) and absorption ($F_{OC,ABS}=-k^{\prime }\times \text{GOC}∕H^{\prime }$), where *H*′ is the dimensionless Henry's law constant and *k*′ is the gas transfer velocity for exchangeable organic carbon estimated from *k*_600_-values and Schmidt numbers assuming an average MW of GOC of 120 g mol^−1^. Thus, negative values of *F*_*OC*_ indicate inputs from the atmosphere into the ocean.

### Back trajectory analysis of air masses

Backward trajectories of the air masses sampled were calculated for the 96 h period prior to the collection of each sample using the HYSPLIT transport and dispersion model provided by the NOAA Air Resources Laboratory (Draxler and Rolph, 2013). The trajectories are shown in Supplementary Figure 1.

### Calculation of mixed layer depth

Mixed layer depth (MLD) was estimated as that presenting a larger than 0.03 kg/m^3^ difference in density from that observed at a reference depth of 10 m (de Boyer Montégut et al., 2004) except for the Arctic samples where the reference depth was 4 m to allow for the shallower MLDs observed in depth profiles of temperature and density.

### Experimental setup

GOC use was evaluated by means of dilution cultures established using the *in situ* prokaryotic community filtered through 0.8 μm polycarbonate filters in order to exclude most grazers and primary producers. GOC was collected upwind of the research vessel, by bubbling air for 1 h at 4 L per min through two separate 1 L gas-washing bottles equipped with a sintered glass diffuser and containing 0.9 L of surface seawater previously filtered through 0.2 μm-pore-size polycarbonate filters (Millipore). In order to avoid potential contamination from the pumping equipment, the air was forced into the collection system by vacuum and a filter (Whatman GF/F) was placed at the inlet to avoid atmospheric dust interference. Duplicate controls were prepared by bubbling high purity synthetic air for 1 h at 4 L per min through 1 L glass-washing bottles containing seawater samples identical to those used for GOC collection, thereby purging the ambient GOC already present in the samples. Immediately after bubbling, the bottles were inoculated with 100 mL of 0.8 μm-filtered surface seawater, closed with airtight screw caps and stored in the dark submersed in on-deck incubators flowed continuously with surface seawater. Prokaryotic growth was checked every day by following changes in prokaryotic abundance by flow cytometry of SYTO 13 stained samples (del Giorgio et al., 1996) previously fixed for 30 min with PBS buffered paraformaldehyde (1% final concentration) and processed in the same day. Both treatments and controls were kept in the dark in tightly closed 1 L bottles at *in situ* temperature until exponential growth was detected in the cultures (between 3 and 6 days after inoculation). At this point samples were withdrawn to estimate prokaryotic respiration and secondary production (*P*) by ^3^H-leucine incorporation (Smith and Azam, 1992). ^3^H-Leucine incorporation (40 nM final concentration) was determined using triplicate live subsamples and duplicate blanks killed by adding trichloroacetic acid (5% final concentration) in 1.2 mL samples using the microcentrifugation method (Smith and Azam, 1992). Prokaryotic respiration (*R*) was measured by determining the consumption of dissolved oxygen in four to five, depending on experiments, replicate 30 mL Winkler bottles incubated for 24 h in the dark at the experimental temperature-relative to oxygen concentrations determined in three replicated initial Winkler bottles. Dissolved oxygen measurements were determined by high precision Winkler titration using a potentiometric electrode with automated end point detection (Mettler Toledo, DL28 titrator), and the rates were converted into C units assuming a respiratory quotient of 1. ^3^H-leucine incorporation was measured twice for every experiment, once at the start of the *R* incubations and once again 24 h later coinciding with the end of *R* incubations. Rates of ^3^H-leucine incorporation integrated over the 24 h interval used for *R* measurements were converted to *P* assuming a conservative leucine to carbon conversion factor of 1.55 kg C mol^−1^ leucine assuming no intracellular dilution (Simon and Azam, 1989).

### Prokaryotic GOC utilization estimates

Prokaryotic growth efficiency (GE) was calculated from prokaryotic production and respiration as the percentage of the total prokaryotic carbon demand (*R* + *P*) allocated to build new biomass:

$$\begin{array}{l} GE=100\;\times \;\frac{P}{\left(P+R\right)} \end{array}$$

GE was always higher in the presence of GOC as compared to purged controls, allowing us to calculate a minimum estimate of GOC utilization in our experiments. The non-volatile fraction of DOM was the same for both treatment and controls. Therefore, it was safe to assume that the non-volatile fraction of the DOC was used with the same efficiency in both the GOC-containing bottles and the GOC-free controls. Under this assumption, the maximum amount of non-volatile DOC consumed was constrained using the observed respiration values. We assumed that all of the respiration measured in the GOC containing bottles was due to non-volatile DOC use, and that non-volatile DOC was used with the same efficiency as in the controls. Thus, at least the part of the prokaryotic production in the GOC containing bottles that could not be explained by growth on the non-volatile DOC pool had to be supported by GOC use. We estimated GOC utilization rates as the proportion of *P* not matched by *R*, assuming that all of the *R* in the GOC containing bottles was supported by non-volatile DOC carbon use. Thus, non-volatile carbon use efficiency for every experiment was determined as the average of the GE observed in the purged controls (*GE*_*c*_). Then we estimated the maximal fraction of *P* attributable to non-volatile carbon utilization (*P*_*nvdoc*_) in the GOC-containing bottles assuming that all of the observed *R* was supported by non-volatile DOC use, using the expression,

$$\begin{array}{l} P_{nvdoc}=R\times \frac{\frac{GE_{c}}{100}}{1-\frac{GE_{c}}{100}} \end{array}$$

which is equivalent to,

$$\begin{array}{l} P_{nvdoc}=R\times \frac{P_{c}}{R_{c}} \end{array}$$

where *P*_*c*_ and *R*_*c*_ are the production and respiration values observed in the purged controls.

The remainder of the production in the GOC containing treatments (*P* − *P*_*nvdoc*_) was then taken as an estimate of GOC utilization rates. These calculations assume that none of the GOC taken up by bacteria is respired, and therefore, the values reported here are minimum estimates of bacterial GOC use. It could be that a large fraction of the GOC was respired as it has been reported for methanol in oligotrophic stations of the tropical Atlantic (Dixon et al., 2010, 2011; Dixon and Nightingale, 2012). In that case GOC utilization rates would be much higher than this minimum estimate since the amount of carbon dedicated to respiration is usually much higher than that dedicated to production (Del Giorgio and Williams, 2005). Since GOC utilization rates derived from the minimum estimate approaches or even exceeds diffusive inputs of GOC at most stations (**Table 2**), the upper limit of GOC use would be constrained by the rates of GOC deposition into surface waters.

### Ectoenzyme activity

Ectoenzymatic activities were measured by means of fluorogenic substrates (Hoppe, 1993) using the procedures described previously (Sala et al., 2010). Briefly, the activities of 9 ectoenzymes were determined using the following substrates: 4-methylumbelliferyl-β-D-glucoside (β-D-glucosidase, BGL), 4-methylumbelliferyl-α-D-glucoside (α-D-glucosidase, AGL), 4-methylumbelliferyl-β-D-xyloside (β-D-xylosidase, XYL), 4-methylumbelliferyl-N-acetyl-β-D-glucosaminide (chitobiase, CHI), 4-methylumbelliferyl-α-L-arabinoside (α-L-arabinosidase, ARA), 4-methylumbelliferyl-β-D-cellobiose (β-D-cellobiohydrolase, CEL), 4-methylumbelliferyl-butyrate (butyrate esterase, BUT), 4-methylumbelliferyl-acetate (acetate esterase, ACE), and L-leucyl-7-amido-4-methylcoumarine (L-leucyl aminopeptidase, AMP). A final concentration of 100 μM of the substrates was added to 0.9 ml samples obtained from the experiment and incubated for between 15 min and 4 h depending on the substrate. Fluorescence was measured before and after the incubation on a Turner Designs, model 10-005-R fluorometer (365/446 nm excitation/emission wavelengths). The increase of fluorescence measured during incubation was converted to hydrolysis rates using standard curves prepared with known quantities of 4-methylumbelliferone for all the substrates except for L-leucyl-7-amido-4-methylcoumarine, which releases 7-amino-4-methylcoumarin upon enzymatic hydrolysis.

Specific cell activity was calculated by dividing ectoenzyme activity by prokaryotic cell abundance measured by flow cytometry.

## Results

### GOC concentrations and estimates of Air-sea GOC exchange

*In situ* concentrations of GOC in water equilibrated with ambient air varied largely among different locations ranging from 5 to 88 μmol C L^−1^ (Table 1) resulting in an average GOC concentration of 37.8 μmol C L^−1^, comparable to previously reported values (Dachs et al., 2005). The initial GOC concentrations accounted for 5–56% of the total DOC (non-purgeable DOC + GOC) present in the GOC treatments (Table 1). No significant differences were detected in the concentrations of phosphorus or nitrogen including ammonium between the GOC containing bottles and the purged controls.

**Table 1:** **Estimates of GOC deposition**.

**Location**	**Date**	**Non-purgeable DOC in seawater (μM C)**	EDOC^*^ (μM C)	***k*_600_**	**Wind speed (m s^−1^)**	**GOC/H′ in seawater (μM C)**	Air-sea OC flux^**^ (mmol C m^−2^ d^−1^)
Mediterranean Sea (A)	04-Jun-06	**71.8**	**24**	**1.23**	**4.5**	**51**	**−25.4**
NE Subtropical Atlantic (B)	21-Aug-06	**79**	**14**	**3.8**	**8**	**19**	**−14.18**
NE Subtropical Atlantic (C)	25-Aug-06	**86.23**	**22**	**3.8**	**8**	**35**	**−36.86**
NE Subtropical Atlantic (D)	03-Feb-07	**68.3**	**73**	**4.29**	**8.5**	**88**	**−47.93**
NE Subtropical Atlantic (E)	19-Feb-07	**82.9**	**18**	**2.92**	**7**	**28**	**−21.81**
Arctic Ocean (F)	05-Jul-07	**94.3**	**35**	**0.98**	**4**	**39**	**−1.62**
Arctic Ocean (G)	20-Jul-07	**91.3**	**0**	**2.16**	**6**	**5**	**−4.21**
		**82.9**	**22**	**2.92**	**7**	**35**	**−18**

* EDOC is defined as the concentration of organic carbon in surface water that can be exchanged with the atmosphere.

** Negative values indicate net inputs from the atmosphere into the ocean. Net fluxes of OC calculated using alternative parameterisations of k_600_ are reported as Supplementary Material.

The amount of exchangeable DOC in surface waters varied between 0 and 73 μmol C L^−1^ representing up to 52% (average 22%) of the total DOC+EDOC pool in surfaces waters.

GOC fluxes were estimated from wind speed and the measured concentrations of EDOC in surface waters and GOC concentrations in water equilibrated with ambient air (GOC/H′). Estimated GOC fluxes were always negative (into the ocean) at all the locations in this study (Table 1), regardless of the parameterisation used to compute the mass transfer velocity (Supplementary materials).

### Prokaryotic production and respiration

**Figure 2:**
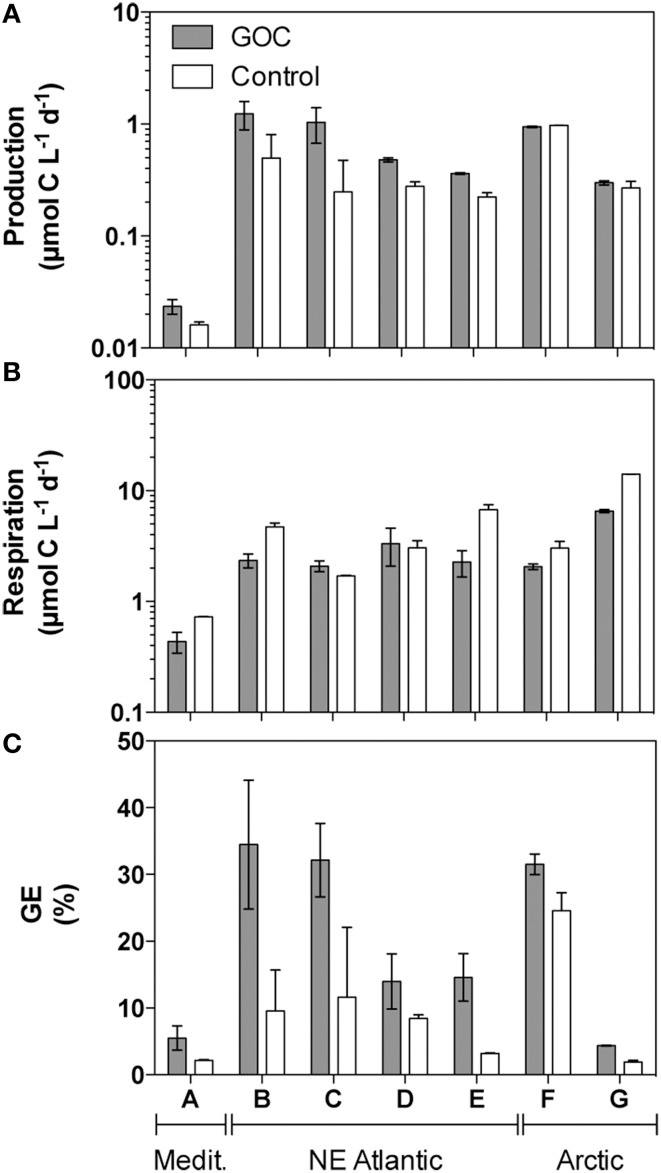
**Prokaryotic production (*P*, panel A), respiration (*R*, panel B), and Growth Efficiency (GE, panel C) in microcosms containing natural GOC (filled bars) vs. GOC-free controls (open bars)**. Bars represent the average value and the error bars represent the range (minimum and maximum) for duplicate microcosms. Letters on the x-axis indicate the origin of the sample as in Table 1. Please note the logarithmic scale on panels **(A, B)**.

Prokaryotic utilization of GOC vs. the non-volatile components of the marine dissolved organic pool was evaluated by comparing the rates of prokaryotic growth and respiration in surface seawater previously equilibrated with ambient levels of GOC vs. controls equilibrated with clean synthetic air. Prokaryotic production (*P*) increased substantially in the presence of GOC as compared to controls in the samples taken in the Mediterranean and subtropical NE Atlantic, but not in those collected in the Arctic (Figure 2A). In general, prokaryotic growth was higher (Wilcoxon matched pair test *p* < 0.05, Figure 2A) in the presence of GOC as compared to the GOC-free controls by about 66% (median value).

In contrast, respiration was often lower (by 38%, median value) in the GOC treatments than in the controls (Figure 2B), but more markedly so in the Arctic samples, where little effect was observed on production. Consequently, prokaryotic growth efficiencies were always higher (Figure 2C, Wilcoxon matched pair test *p* < 0.05) in the presence of GOC as compared to GOC-free controls. In summary, marine prokaryotes showed 2.5 times (median) higher growth per unit organic carbon processed when growing in the presence of atmospheric carbon than when using non-purgeable DOC alone.

### Ectoenzyme activity

**Figure 3:**
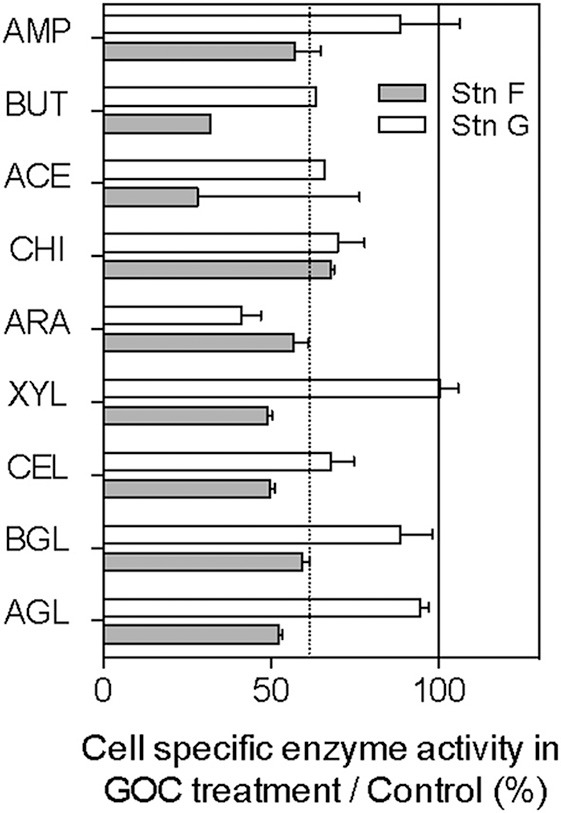
**Cell-specific enzyme activity in GOC treatment as percentage of the values observed in the controls**. The dotted line shows the median value and the continuous line shows the 100% line indicating no effect of GOC. Hydrolytic enzyme activities were measured only in the Arctic stations F and G. AMP, L-leucyl aminopeptidase; BUT, butyrate esterase, ACE, acetate esterase; CHI, chitobiase; ARA, α-L-arabinosidase; XYL, β-D-xylosidase; CEL, cellobiosidase; BGL, β-D-glucosidase; and AGL, α-D-glucosidase.

We measured ectoenzyme activity in the two experiments carried out in the Arctic (stations F and G, Figure 3) targeting nine enzymes responsible for the extracellular hydrolysis of carbohydrates, lipid esters, and proteins. The majority of the ectoenzymes tested showed substantially lower activity in the presence of GOC (Figure 3) as compared to the controls, resulting in a 37% average reduction of hydrolytic activity in the GOC treatments (Figure 3).

### Prokaryotic consumption of GOC

Total prokaryotic utilization of GOC was estimated as the fraction of prokaryotic production in the GOC treatments that could not be explained using the growth efficiencies observed in the controls (see Section Materials and Methods for an extended description). According to this conservative estimate, atmospheric-derived compounds provided between 2 and 27% of the total prokaryotic carbon demand in surface ocean waters (Table 2).

**Table 2:** **Estimates of microbial utilization of GOC**.

**Location**	**GOC treatment *P*, mmol C m^−3^ d^−1^**	**GOC treatment *R*, mmol C m^−3^ d^−1^**	**Control *P*, mmol C m^−3^ d^−1^**	**Control *R*, mmol C m^−3^ d^−1^**	**Minimum % of prokaryotic carbon demand supported by GOC**	Integrated GOC consumption^*^, mmol C m^−2^ d^−1^	% Incoming GOC utilized^*^
Mediterranean Sea (A)	**0.023**	**0.434**	**0.016**	**0.728**	**3.40**	**0.22**	**0.87**
	0.020–0.027	0.342–0.526	0.015–0.017	0.726–0.731	1.54–5.26	0.13–0.31	0.53–1.22
NE Subtropical Atlantic (B)	**1.236**	**2.348**	**0.496**	**4.727**	**27.18**	**24.66**	**173.91**
	0.888–1.585	2.010–2.687	0.184–0.808	4.345–5.109	16.49–37.88	14.90–34.42	105.06–242.77
NE Subtropical Atlantic (C)	**1.038**	**2.088**	**0.248**	**1.702**	**22.14**	**16.95**	**45.98**
	0.674–1.401	1.857–2.320	0.021–0.475	1.680–1.725	15.82–28.46	9.30–24.60	25.23–66.73
NE Subtropical Atlantic (D)	**0.480**	**3.329**	**0.278**	**3.051**	**6.04**	**23.68**	**49.41**
	0.460–0.500	2.078–4.580	0.252–0.304	2.554–3.547	1.53–10.55	10.63–36.74	22.18–76.64
NE Subtropical Atlantic (E)	**0.361**	**2.266**	**0.223**	**6.738**	**11.74**	**24.39**	**111.84**
	0.355–0.368	1.660–2.871	0.202–0.243	5.990–7.487	8.06–15.42	22.15–26.64	101.54–122.14
Arctic Ocean (F)	**0.945**	**2.058**	**0.972**	**3.034**	**9.12**	**1.53**	**94.35**
	0.933–0.958	1.939–2.176	0.968–0.976	2.583–3.485	7.07–11.17	1.24–1.82	76.36–112.34
Arctic Ocean (G)	**0.299**	**6.538**	**0.268**	**14.047**	**2.55**	**1.46**	**34.71**
	0.287–0.312	6.333–6.744	0.229–0.308	13.978–14.115	2.50–2.59	1.39–1.53	33.00–36.42
	**5.76**	**25.5**	**2.97**	**42.19**	**9.3**	**9.97**	**71.54**

Values indicate the average (bold) and range of duplicate estimates. The bottom row shows the median values for each variable.

* Extending prokaryotic utilization to the entire depth of the mixed layer.

## Discussion

The presence of GOC resulted in substantial changes in prokaryotic C demand in all the experiments as compared to GOC-free controls. Prokaryotic production was enhanced by GOC in the Mediterranean and in the subtropical NE Atlantic indicating that growth on non-volatile DOC was probably limited by the availability of labile carbon in these samples, while little influence on prokaryotic production was detected in the more productive Arctic samples. The largest effects of GOC on prokaryotic production were observed in the samples taken in summer in the subtropical NE when primary productivity is known to be at its minimum for this region (Teira et al., 2005). Respiration rates never matched the increase in prokaryotic production observed in the presence of GOC. Instead, GOC containing treatments often showed much lower respiration rates than the corresponding controls resulting in much higher growth efficiency when GOC was available. Since the non-volatile DOC fraction was the same in both the GOC containing bottles and the controls, this indicates that GOC supported much higher growth efficiencies than non-volatile DOC. However, earlier considerations of the possible role of atmospheric inputs of organic material assumed that GOC consists of poor substrates supporting low growth efficiencies and therefore, that GOC was used only in unproductive systems with a limited supply of labile organic carbon (del Giorgio and Cole, 1998). Yet, no estimates of GOC use in the ocean were available until now to support this expectation. Conversely, our results show widespread use of GOC/*H*′ supporting high prokaryotic growth efficiencies across all the oceanic regions investigated. Prokaryotic growth efficiency on small molecules is largely determined by the degree of reduction of the substrate, with maximum growth yields expected for molecules more reduced than microbial biomass (Linton and Stephenson, 1978; Gommers et al., 1988; Vallino et al., 1996; Babel, 2009). As stated before, GOC is a complex mixture containing many unidentified components but the major characterisable components of atmospheric GOC such as methanol, acetone, and acetaldehyde present degrees of reduction higher than that of microbial biomass, which may result in high growth efficiencies. Relatively high growth efficiencies >20% have been measured for methanol utilization in surface waters of the NE Atlantic although much lower efficiencies have also been reported at some other locations (Dixon et al., 2010, 2011; Dixon and Nightingale, 2012). Methanol alone has been found to cover on average about 13% of the total microbial carbon demand and up to 50% of prokaryotic production in the Subtropical Atlantic with a turnover as low as 1 day (Dixon et al., 2011). Yet, there are many other biogenic and anthropogenic semi-volatile organic compounds in the gas phase with a high tendency to deposit in the ocean contributing to the GOC pool (Goldstein and Galbally, 2007; Hauser et al., 2013) including aliphatic and aromatic hydrocarbons, and oxygenated hydrocarbons also presenting degrees of reduction larger than those of microbial biomass. Thus, the available information supports the idea that the identifiable organic constituents of the troposphere can support rapid prokaryotic growth and relatively high growth efficiencies in the surface ocean.

Additionally, we detected inhibition of hydrolytic enzyme activity in the presence of GOC in the Arctic samples. The synthesis and export of extracellular hydrolases constitutes a major energetic expenditure to planktonic bacteria (del Giorgio and Cole, 1998) which can result in up to 30% reduction in growth efficiency (Middelboe and Søndergaard, 1993). Thus, the observed shift from the utilization of macromolecular DOC in the controls to the use of the readily available pool of small molecules characteristic of GOC is consistent with the higher GEs measured in our experiments.

GOC organic compounds-supported between 2 and 27% of the prokaryotic carbon demand. Extending the observed consumption rates to the whole depth of the mixed layer (Table 2) and comparing them to diffusive GOC fluxes calculated independently using the local carbon pools and wind speed (Table 1) would result in removal of between 30 and >100% of the diffusive input of GOC by planktonic prokaryotes (Table 2) with the only exception of station A, located in the Mediterranean Sea, where the estimated consumption was < 1% of the GOC input. The air mass sampled at station A probably carried a large proportion of terrestrial and anthropogenic organics as indicated by the air mass back trajectory analysis (Supplementary Figure 1), which could explain the low utilization values observed.

The ultimate origin of the atmospheric GOC compounds in our samples is uncertain. It may have been produced in the ocean, or could be the result of volatile compounds that are emitted from the ocean, and after atmospheric oxidation produce compounds with lower *H*′-values (semi-volatile), thus with higher tendency to be deposited through diffusive absorption. Indeed, back trajectory analysis of air masses indicates a marine origin of GOC at all the stations sampled except for station A (Supplementary Figure 1). Yet, the large distances traveled by the sampled air masses over the 96 h period modeled suggest that a large fraction of the GOC was produced at remote locations. Thus, long-range transport of bioavailable GOC could help explain local observations of heterotrophy in some regions of the oceans, including the subtropical NE Atlantic investigated here (Duarte et al., 2013). The ultimate origin of the GOC components exchanged between the ocean and the atmosphere is an important issue that demands further research. However, whether or not GOC compounds where produced originally in the ocean does not change our finding that natural marine prokaryotic communities can use efficiently the pool of GOC entering the ocean from the atmosphere.

Evidence of significant prokaryote utilization of diffusive organic inputs spread over the Mediterranean, the North Atlantic and the Atlantic sector of the Arctic Ocean indicates that this is a pervasive process and warrants further investigation. Present depictions of the role of the oceans in the global C cycle assume that the oceans exchange only CO_2_ with the atmosphere, only accounting for the air–sea exchange of few organic compounds. However, our data indicate that organic molecules exchanged between the ocean and the atmosphere represent a significant fraction of the total DOC in surface waters and, more importantly, of the bioavailable DOC pool which has been systematically ignored. Extrapolating our limited dataset to the global ocean involves considerable uncertainty, since the measured rates may not represent adequately the magnitude of GOC utilization in other oceans or the temporal dynamics associated to the seasonality of gaseous organic emissions. Yet, our estimates of GOC utilization over the mixed layer range from 0.2 to 24.3 mmol C m^−2^ d^−1^ (Table 2), a magnitude comparable to the average uptake of CO_2_ of 1.3 mmol C m^−2^ d^−1^ reported for the global ocean (Takahashi et al., 2009). Moreover, the global carbon budget of the ocean is based almost entirely on measurements of the non-purgeable, components of DOC since the most common methods used to measure oceanic DOC exclude the volatile components (Spyres et al., 2000). Our results have implications for *in vitro* studies of microbial activity and DOC utilization where volatile and semi-volatile compounds can be excluded by vacuum-filtration procedures, possibly leading to artifacts in the estimation of microbial activity in the field. Thus, including GOC and EDOC in the global DOC estimates may help filling some of the obvious gaps presently found in the oceanic carbon budget.

In summary, our results indicate that prokaryotic use of GOC is a prevalent process, accounting for a significant part of the prokaryotic carbon demand in surface waters across contrasting oceanic regions. In addition, prokaryotic consumption of GOC is likely to have a large impact on the global carbon cycle by affecting the rates and even the direction of the fluxes of organic materials between the ocean and the atmosphere. Incorporating both deposition and utilization of GOC into the carbon budget of the global ocean is a matter of urgency, as the results presented here suggest that the ocean may be a much larger sink of atmospheric organic materials than hitherto recognized.

## Author contributions

JA and CD designed the experiments, analyzed the data and wrote the manuscript. JA implemented the experimental setup and measured prokaryotic abundance and production. CD measured respiration, DOC and nutrients. CD and JD measured GOC concentrations and estimated the diffusive fluxes of GOC. MS measured enzymatic activity. All authors discussed the results and commented on the manuscript.

## Funding

This is a contribution to projects RODA (CTM2004-06842-CO3-02), and ATOS (POL2006-00550/CTM) projects, funded by the Spanish Ministry of Science and Innovation and project THRESHOLDS funded by the 6^th^ Framework Programme of the European Union. JA was supported by a “Ramón y Cajal” research fellowship from the Spanish Government.

### Conflict of interest statement

The authors declare that the research was conducted in the absence of any commercial or financial relationships that could be construed as a potential conflict of interest.
