# Supplementary material for: Out of Thin Air: Microbial Utilization of Atmospheric Gaseous Organics in the Surface Ocean
Source: Front Microbiol. 2016 Jan 20;6:1566. doi: 10.3389/fmicb.2015.01566 (PMC4718972; doi:10.3389/fmicb.2015.01566)
Supplement: Supplementary file 1 [file DataSheet1.DOC]

Supplementary figure 1. Back trajectory analysis of the air masses sampled. The star represents the sampling point and the red trace, the modelled trajectory of the sampled air mass in the 96 h before sampling. The triangles show the position of the air mass in 6 h steps. The lower pannel of each graph represents the vertical position in meters above ground level (meters AGL) .

**Influence of the parametrisation used to calculate k_600_**

The net flux of organic compounds between the sea surface and the atmosphere in the manuscript was estimated using the following parametrisation of the relationship between gas exchange and wind, $k_{600}=0.24U_{10}^{2}+0.061U_{10}$ ^1^ derived from oceanic measurements, including open-ocean locations.

Other parametrisations have been reported in the relevant literature including simple quadratic relationships,$k_{600}=0.266U_{10}^{2}$ ^2^, $k_{600}=0.27U_{10}^{2}$ ^3^, asymptotic quadratic relationships such as those reported for the North Atlantic $k_{600}=3.3+0.026U_{10}^{2}$ ^4^, or the equatorial Pacific $k_{600}=8.2+0.014U_{10}^{2}$ ^5^ or even third degree polynomials $k_{600}=3+0.1U_{10}+0.064U_{10}^{2}+0.011U_{10}^{3}$ ^6^.

Supplementary figure 2. Net fluxes of OC carbon between the atmosphere and the surface ocean calculated using different parametrisations. Negative numbers indicate that the direction of the flux was into the ocean.

Thus, we calculated net fluxes of organic compounds in our experiments using the above-mentioned functions in order to constrain our estimates. Net fluxes were always negative (into the ocean) regardless of the parameterisation used. The estimates reported in the manuscript (red dots in Supplementary figure 2). Moreover, most of the parameterisations except for the polynomial approach ^6^ yielded higher fluxes than the one we used in this study or sometimes slightly (1 to 6%) lower fluxes (Supplementary table 1). Most parameterisations showed good agreement with differences in the range of <10% as compared the formulation used in the manuscript. However one of the parameterisations ^5^ resulted in high percentual increases in flux at low wind speeds (4-6 m s^-1^). Only the parameterisation of Wanninkhof et al. ^6^ yielded consistently lower fluxes by about 15% at higher wind speeds (>6 m s^-1^) as described in the original paper. The difference between the value given by the parameterisation of Nightingale et al. ^1^ used in this study and the median value of the other parameterisations is ≤10% for all the stations except for station F (22%). However, large deviations at station F are not surprising since the value of the net flux is small for this station and thus small variations in net flux result in high percentual variation. Thus, the choice of parameterisation used to calculate k_600_ is not a major concern in our calculations since it is in the range of other parameterisations for wind speeds >6 m s^-1^ and quite a conservative estimate for slower winds where the different parameterisations show larger differences ^6^. The parameterisation of Nightingale et al. ^1^ was chosen for further calculations as it resulted in intermediate, near average values of net flux for most of the stations (Supplementary figure 2).

Supplementary table 1. Magnitude of the net flux of OC carbon between the atmosphere and the surface ocean calculated using different parametrisations (mmol C m^-2^ d^-1^). Negative fluxes indicate flux into the ocean. The numbers indicated in columns 4-8 indicate whether the number was higher (positive black numbers) or lower (negative red numbers) than the flux calculated using the approximation of Nightingale et al. ^1^ (column 3) used in the body of the manuscript.

| **Station** | **windspeed**  **(m s^-1^)** | **Nightingale et al. 2000** | **Ho et al. 2006** | **Sweeney et al. 2007** | **McGills et al. 2001** | **McGills et al. 2004** | **Wanninkhof et al. 2009** |
| --- | --- | --- | --- | --- | --- | --- | --- |
| **A** | 4.5 | **-25.44** | **-26.69**  +5% | **-27.09**  +6% | **-28.09**  +10% | **-46.95**  +85% | **-28.48**  +12% |
| **B** | 8 | -**14.18** | **-15.23**  +7% | **-15.46**  +8% | **-14.86**  +5% | **-13.75**  -3% | **-12.1**  -15% |
| **C** | 8 | **-36.86** | **-39.6**  +7% | **-40.2**  +8% | **-38.64**  +5% | **-35.75**  -3% | **-31.47**  -15% |
| **D** | 8.5 | **-47.93** | **-51.58**  +8% | **-52.36**  +9% | **-51.71**  +8% | **-45.08**  -6% | **-40.88**  -15% |
| **E** | 7 | **-21.81** | **-23.32**  +7% | **-23.67**  +8% | **-21.86**  +0.3% | **-23.26**  +7% | **-18.98**  -13% |
| **F** | 4 | **-1.62** | **-1.69**  +4% | **-1.71**  +6% | **-1.97**  +22% | **-3.61**  +123% | **-2.03**  +26% |
| **G** | 6 | **-4.21** | **-4.48**  +6% | **-4.54**  +7% | **-4.17**  -1% | **-5.25**  +25% | **-3.87**  -8% |

**References**

1. Nightingale, P. D. *et al.* In situ evaluation of air-sea gas exchange parameterizations using novel conservative and volatile tracers. *Global Biogeochem. Cycles* **14,** 373–387 (2000).

2. Ho, D. T. *et al.* Measurements of air-sea gas exchange at high wind speeds in the Southern Ocean: Implications for global parameterizations. *Geophys. Res. Lett.* **33,** L16611 (2006).

3. Sweeney, C. *et al.* Constraining global air-sea gas exchange for CO2 with recent bomb 14C measurements. *Global Biogeochem. Cycles* **21,** GB2015 (2007).

4. McGillis, W. R., Edson, J. B., Hare, J. E. & Fairall, C. W. Direct covariance air-sea CO 2 fluxes. *J. Geophys. Res.* **108,** 16 (2001).

5. McGillis, W. R. *et al.* Air-sea CO2 exchange in the equatorial Pacific. *J. Geophys. Res.* **109,** C08S02 (2004).

6. Wanninkhof, R., Asher, W. E., Ho, D. T., Sweeney, C. & McGillis, W. R. Advances in Quantifying Air-Sea Gas Exchange and Environmental Forcing*. *Annual Review of Marine Science* **1,** 213–244 (2009).
